# Supplementary material for: Identification and functional analysis of non-coding regulatory small RNA FenSr3 in Bacillus amyloliquefaciens LPB-18
Source: PeerJ. 2023 May 15;11:e15236. doi: 10.7717/peerj.15236 (PMC10194069; doi:10.7717/peerj.15236)
Supplement: Supplemental Information 4 [file peerj-11-15236-s004.zip › KO/CK-vs-T1_map/map04923.html]

KEGG PATHWAY: Regulation of lipolysis in adipocytes - Reference pathway


|  |  |
| --- | --- |
| **Regulation of lipolysis in adipocytes - Reference pathway** |  |

[
Pathway menu
| Organism menu
| Pathway entry
| Show description
| User data mapping
]

Lipolysis in adipocytes, the hydrolysis of triacylglycerol (TAG) to release fatty acids (FAs) and glycerol for use by other organs as energy substrates, is a unique function of white adipose tissue. Lipolysis is under tight hormonal control. During fasting, catecholamines, by binding to Gs-coupled-adrenergic receptors (-AR), activate adenylate cyclase (AC) to increase cAMP and activate protein kinase A (PKA). PKA phosphorylates target protein such as hormone-sensitive lipase (HSL) and perilipin 1 (PLIN). PLIN phosphorylation is a key event in the sequential activation of TAG hydrolysis involving adipose triglyceride lipase (ATGL), HSL, and monoglyceride lipase (MGL). During the fed state, insulin, through activation of phosphodiesterase-3B (PDE-3B), inhibits catecholamine-induced lipolysis via the degradation of cAMP.

|  |  |  |
| --- | --- | --- |
| Reference pathway | 184% 150% 122% 100% 82% 67% 55% | 图片下载 |
